# Clarifying the concept of a learning health system for healthcare delivery organizations: Implications from a qualitative analysis of the scientific literature

**DOI:** 10.1002/lrh2.10287

**Published:** 2021-07-22

**Authors:** Douglas Easterling, Anna C. Perry, Rachel Woodside, Tanha Patel, Sabina B. Gesell

**Affiliations:** ^1^ Department of Social Sciences and Health Policy Wake Forest School of Medicine Winston‐Salem North Carolina USA; ^2^ Wake Forest Clinical and Translational Science Institute, Wake Forest School of Medicine Winston‐Salem North Carolina USA; ^3^ North Carolina Translational and Clinical Sciences Institute University of North Carolina School of Medicine Chapel Hill North Carolina USA

**Keywords:** consolidated framework, learning health system, learning healthcare system

## Abstract

The “learning health system” (LHS) concept has been defined in broad terms, which makes it challenging for health system leaders to determine exactly what is required to transform their organization into an LHS. This study provides a conceptual map of the LHS landscape by identifying the activities, principles, tools, and conditions that LHS researchers have associated with the concept. Through a multi‐step screening process, two researchers identified 79 publications from PubMed (published before January 2020) that contained information relevant to the question, “What work is required of a healthcare organization that is operating as an LHS?” Those publications were coded as to whether or not they referenced each of 94 LHS elements in the taxonomy developed by the study team. This taxonomy, named the Learning Health Systems Consolidated Framework (LHS‐CF), organizes the elements into five “bodies of work” (organizational learning, translation of evidence into practice, building knowledge, analyzing clinical data, and engaging stakeholders) and four “enabling conditions” (workforce skilled for LHS work, data systems and informatics technology in place, organization invests resources in LHS work, and supportive organizational culture). We report the frequency that each of the 94 elements was referenced across the 79 publications. The four most referenced elements were: “organization builds knowledge or evidence,” “quality improvement practices are standard practice,” “patients and family members are actively engaged,” and “organizational culture emphasizes and supports learning.” By dissecting the LHS construct into its component elements, the LHS‐CF taxonomy can serve as a useful tool for LHS researchers and practitioners in defining the aspects of LHS they are addressing. By assessing how often each element is referenced in the literature, the study provides guidance to health system leaders as to how their organization needs to evolve in order to become an LHS ‐ while also recognizing that each organization should emphasize elements that are most aligned with their mission and goals.

## 1 INTRODUCTION

Leading health institutions such as the National Academy of Medicine (NAM), the National Institutes of Health (NIH), and the Agency for Healthcare Research and Quality (AHRQ) are encouraging healthcare organizations to become “learning health systems” or “learning healthcare systems” (LHS) as a means of accelerating both the translation of research into practice and the development of interventions that will improve patient care and patient outcomes.^1^, ^2^, ^3^ The LHS concept calls for healthcare organizations to be more systematic and data‐driven in generating and utilizing knowledge to improve the quality and value of the care they deliver, while also stimulating innovation.^4^

The following definitions of LHS were offered by the Institute of Medicine (IOM) in 2007 and 2013, respectively:

- An LHS *“generates and applies the best evidence for the collaborative healthcare choices of each patient and provider; drives the process of discovery as a natural outgrowth of patient care, and ensures innovation, quality, safety and value in health care.”* (ix)^5^
- “*In a learning healthcare system, science, informatics, incentives and culture are aligned for continuous improvement and innovation, with best practices seamlessly embedded in the delivery process, patients and families active participants in all elements, and new knowledge captured as an integral by‐product of the delivery experience.” (136)* ^6^

The 2013 definition has been actively promoted by IOM and its successor, NAM. It is highlighted in the charter of NAM's Leadership Consortium: Collaboration for a Value and Science‐Driven Health System.^7^

AHRQ released a similar, although less detailed definition of LHS in 2019:

*“In a learning health system, internal data and experience are systematically integrated with external evidence, and that knowledge is put into practice. As a result, patients get higher quality, safer, more efficient care, and health care delivery organizations become better places to work*.” (1)^8^ ^.^

By design, these definitions are broad and aspirational, leaving out the details that would guide healthcare organizations on the specific work they should be carrying out. According to the 2007 IOM report, LHS is purposefully presented as a broad definition, which can be adapted to different contexts.^5^ In turn, this has allowed ‐ and even encouraged ‐ a panoply of diverse activities, tools, and principles to be associated with the LHS concept. Different elements are emphasized by the different subfields that have embraced LHS, including health services research, systems science, organizational theory, clinical informatics, implementation science, and quality improvement. This diversity in how LHS is described makes it challenging for health system leaders to know what is actually required of their organization to qualify as an LHS. We believe that a comprehensive taxonomy of LHS elements will be useful to health system leaders in understanding what LHS has come to mean, which is an important first step in determining, which forms of work should be emphasized as their own organizations implement the LHS concept.

### 1.1 Study aims

The current study uses a targeted review and analysis of relevant scientific literature to identify the various elements that have been associated with operating as an LHS and to provide a sense of which elements are emphasized by LHS researchers. The key product of the study is a comprehensive taxonomy accompanied by frequency counts for each element. The study focused specifically on publications where authors described what healthcare organizations are doing ‐ or should be doing ‐ in accord with the LHS concept. Publications meeting this criterion were reviewed by qualitative researchers who identified text passages that communicated how the authors were conceptualizing LHS. That text was then coded according to a taxonomy to determine which elements were present in each publication.

### 1.2 Focus on healthcare organizations

It is important to reiterate that this study is focused on one specific form of LHS, namely individual healthcare organizations that are conducting systematic learning in support of improved patient care. As Guise, Savitz, and Friedman point out, there are other entities (eg, collaborative learning networks, national health systems) that are aiming to act as an LHS.^9^ In addition, the LHS construct has been applied to specific approaches to learning and translating knowledge, such as “Making Sense of Big Data.”^10^ The literature review omitted publications with these alternative frames of reference, although we expect that the practices and conditions associated with LHS organizations have more general applicability for all forms of LHS.

## 2 METHODS

The study involved a review and qualitative analysis of scientific publications that offer a perspective on what it means for a healthcare organization to operate as an LHS. Three distinct tasks were carried out: 1) identification of relevant publications; 2) development of a taxonomy of the principles, practices, tools, and conditions that researchers have associated with acting as a learning health system; and 3) coding of publications according to that taxonomy.

### 2.1 Identification of relevant publications

The search for publications was performed within the PubMed database on January 28, 2020. The following four queries were used in the search field: “learning health system,” “learning health systems,” “learning healthcare system,” and “learning healthcare systems.” All publications returned from these four searches (regardless of publication date) were included in the screening process.

The four PubMed searches returned the following numbers of publications:

- “learning health system” (n = 298),
- “learning health systems” (n = 219),
- “learning healthcare system” (n = 114),
- “learning healthcare systems” (n = 43).

Combining these four sets of publications and eliminating duplicates generated a list of 580 potentially eligible publications.

**Inclusion criteria.** Publications were included in the analysis if they met any of the following criteria: 1) described the practices and organizational characteristics that healthcare organizations should adopt or have in place in order to qualify as an LHS; 2) recommended what healthcare organizations should do to transition to and/or maintain an LHS; 3) recommended a specific learning system or learning cycle for healthcare organizations to adopt; or 4) provided an example of a healthcare organization that is operating as an LHS. To be included in the study, the publication also needed to offer specific guidance that went beyond simply restating the definition of LHS offered by IOM, NAM, or AHRQ.

**FIGURE 1:**
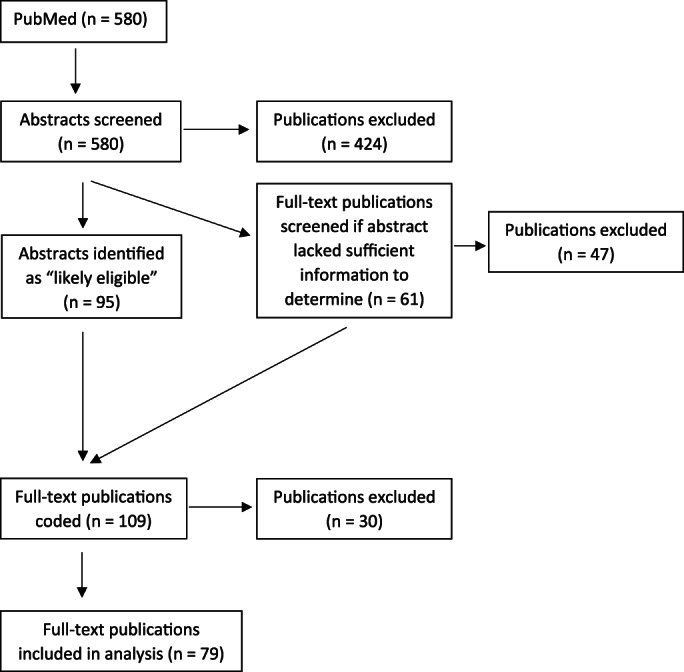
Flow diagram for selecting eligible publications^11^

**Screening process.** The screening process yielded 79 publications that met the eligibility requirements for the study. The flow diagram for the process is shown in Figure 1. The initial step in this process involved reviewing the abstracts for each of the 580 publications identified in the literature search. Two members of the research team independently reviewed each abstract and classified them as either a) appears to meet the eligibility criteria, b) does not meet the criteria, or c) unclear whether the criteria are met. The researchers compared the classifications of each publication and reached consensus. The principal investigator resolved any disagreement. Of the 580 publications, 424 were regarded as not meeting the eligibility requirements and 95 were regarded as “appears to meet the eligibility criteria.” The remaining 61 abstracts lacked sufficient detail to determine eligibility.

For those 61 publications, the researchers independently reviewed the full text, made a determination and compared their classifications. After reviewing the full text, 14 publications that appeared to meet the eligibility criteria advanced to the coding stage of the study. The 109 “appears to meet eligibility criteria” publications were each slated for full‐text coding. During that coding process, the coders determined that 30 of the publications did not meet the eligibility criteria. Thus, the final corpus comprised of 79 publications.^4^, ^9^, ^12^, ^13^, ^14^, ^15^, ^16^, ^17^, ^18^, ^19^, ^20^, ^21^, ^22^, ^23^, ^24^, ^25^, ^26^, ^27^, ^28^, ^29^, ^30^, ^31^, ^32^, ^33^, ^34^, ^35^, ^36^, ^37^, ^38^, ^39^, ^40^, ^41^, ^42^, ^43^, ^44^, ^45^, ^46^, ^47^, ^48^, ^49^, ^50^, ^51^, ^52^, ^53^, ^54^, ^55^, ^56^, ^57^, ^58^, ^59^, ^60^, ^61^, ^62^, ^63^, ^64^, ^65^, ^66^, ^67^, ^68^, ^69^, ^70^, ^71^, ^72^, ^73^, ^74^, ^75^, ^76^, ^77^, ^78^, ^79^, ^80^, ^81^, ^82^, ^83^, ^84^, ^85^, ^86^, ^87^, ^88^

The excluded publications fell into one or more of the following categories: 1) did not devote any attention to LHS in the text, even though LHS was listed as a keyword; 2) made only a passing reference to LHS as a concept somehow related to the focus of the publication; 3) conceptualized LHS narrowly as a particular form of clinical data system rather than a more comprehensive approach to be adopted by healthcare organizations; or 4) conceptualized LHS as a network of organizations – either a national network or a network focused on a particular disease or patient population (eg, PEDSnet) – without describing the work that occurs within individual members of the network.

### 2.2 Development of taxonomy for coding

Selected publications were coded according to a taxonomy that delineates the various principles, practices, tools, and conditions that these publications associate with healthcare organizations operating as an LHS. The taxonomy consists of 94 elements, which relate to topics such as organizational learning, continuous quality improvement, conducting research, clinical data systems, translation of knowledge into practice, engaging stakeholders, skills and training, and organizational policies and culture.

The 94 elements include 38 primary elements and 56 secondary elements. A secondary element is one that provides additional specificity for a primary element. For example, the primary element, “Findings from research are shared or disseminated” has two secondary elements: “Shared internally within the organization” and “Shared externally.”

**Initial development.** The LHS taxonomy was developed through an iterative process, starting with a pilot study, which qualitatively analyzed a limited set of LHS‐related publications. In this pilot study, two librarians at the Coy Carpenter Library at Wake Forest School of Medicine conducted a literature search with a broad lens that included LHS and “organizational learning” within healthcare organizations. The librarians identified 29 publications offering a range of perspectives. The study team reviewed those publications and selected those that provided information on LHS principles and practices. Each of the 12 selected articles was analyzed using NVivo qualitative software.^89^ This analysis gave rise to a draft taxonomy consisting of 19 elements organized under five domains (Learning is a Core Practice, Informatics and Data Systems, Quality Improvement, Engaging Patients and Other Stakeholders, and Context Supportive of Learning).

**Testing and refinement.** This draft taxonomy was applied and expanded during the “testing” phase of the current study. Following the PubMed search and screening process described above, the study team purposefully selected 14 publications offering a diverse range of perspectives on the LHS construct. Two researchers independently coded these 14 articles, while also noting LHS‐relevant text that did not fit any of the existing codes. Coding results were compared and discussed by the two researchers and the principal investigator. This process occurred in three waves. The first wave included review and discussion of codes for the first five publications. The second wave brought in five additional publications and the third wave, the remaining four publications. These meetings included decisions to add missing codes to the taxonomy and to clarify ambiguous codes.

At the end of this testing phase with the 14 publications, the taxonomy had expanded from 19 elements to 85 elements (32 primary and 53 secondary). This expansion resulted from two factors. First, the 14 publications coded in the testing phase included a number of LHS‐related features not mentioned in the 12 publications analyzed during the pilot study. Second, some of the elements generated during the pilot study were deemed to be too broad and/or to connote multiple constructs. Whereas the pilot study provided a first‐cut “unpacking” of the constructs that have been associated with LHS, the testing phase was designed to create a taxonomy of more specific elements that could be used to code text in LHS publications.

One tactic in creating a more fine‐grained taxonomy was to include secondary elements as a means of representing key distinctions and specifications that arose when authors described concepts such as learning processes, dissemination, implementation, and engaging stakeholders. In developing secondary elements for a particular primary element, we sometimes specified a secondary element that we expected to be relevant to publications that would be coded beyond the testing phase. For example, “implements with fidelity” was included as one of five secondary elements for “the organization is systematic in its implementation processes” because we assumed this would be mentioned in at least one publication ‐ although this turned out not to be the case.

The taxonomy evolved modestly (from 85 to 94 elements) as additional publications were reviewed and coded during the analysis phase. When coders encountered LHS‐relevant text that did not fit the existing taxonomy, they flagged the text and brought it up for discussion and resolution in the weekly conversations between the two coders and the principal investigator. Those meetings also led to further clarification of the coding rules.

The 14 publications used to develop the taxonomy were included in the main analysis. Because the taxonomy expanded during the testing phase, these 14 publications were re‐coded according to the revised taxonomy.

### 2.3 Coding of publications

The intent of the coding process was to determine, which elements of the LHS construct were mentioned in each publication. Two researchers reviewed and coded each of the publications that met the inclusion criteria. The coding process involved identifying passages pertaining to the conceptualization of LHS and then assigning those passages to the appropriate item within the LHS taxonomy. A codebook (available upon request) was created with rules for when to apply a particular LHS element.

The coding process was performed using Dedoose software for qualitative analysis.^90^ The two coders performed their coding independently. Approximately once per week, they met along with the principal investigator to compare how they coded the most recent batch of 5‐10 publications. All discrepancies were discussed and reconciled.

As an indication of how much reconciliation was required, we assessed inter‐rater agreement in coding prior to the reconciliation meetings (ie, when the coders were operating independently). This involved the calculation of Cohen's Kappa for a sample of 10 publications at roughly the mid‐point of coding process. The practical question behind this calculation was whether there was enough agreement between the coders to move toward single‐person coding. The observed Kappa was 0.705, indicating “moderate” agreement.^91^ Based on this result, the study team determined that due to the complexity of the coding task, it was essential to continue with the procedure of dual‐coding and facilitated meetings to reconcile differences.

In summary, each step of the process, including taxonomy development, screening, and coding, was completed by two members of the research team. One individual was consistent for all of these steps, while the second individual changed for each step in the process.

## 3 RESULTS

### 3.1 Sample of publications

The 79 publications in our sample were published by 50 different journals, plus National Academies Press (NAP). The journal with the highest representation was *Learning Health Systems* with 14 publications (or 18%). The *Journal of Comparative Effectiveness* published four of the articles in the sample, while NAP published four of the books in the sample. No other journal had more than three articles represented in the sample.

**FIGURE 2:**
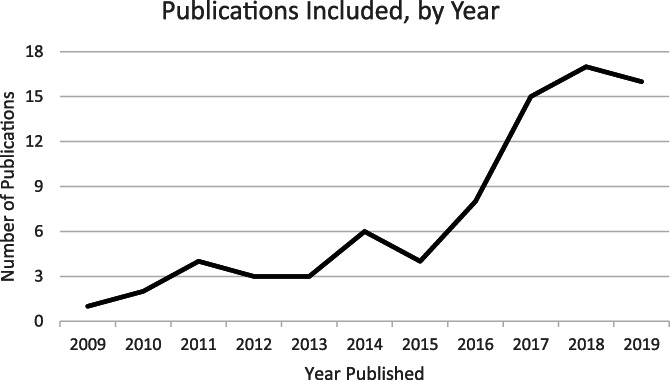
Publications included in the qualitative analysis of the literature, by year of publication. *Note *: January 2020 publications are included in the year 2019

The 79 articles/books were published between 2009 and January 2020 (which is when the search was performed). Publication dates are plotted in Figure 2. The figure shows a dramatic uptick in LHS publications beginning in 2016.

The publications referenced a variety of healthcare organizations when presenting LHS research findings and making recommendations for LHS work. Fourteen of the publications (18% of the sample) referenced an academic health system, while seven (9%) referenced a non‐academic health system. The highest representation was Nationwide Children's Hospital with three publications. In addition to these 21 publications that referenced particular healthcare organizations, the sample included 8 (10%) publications that referenced both academic and non‐academic health systems, and 6 (8%) that referred to a national network of healthcare organizations. Over half of the publications (44, or 56%) did not explicitly identify the type of organization that served as the context for their study or recommendations.

### 3.2 Coding of each publication

The coding process generated an “LHS profile” for each publication, where each of the 94 elements in the taxonomy was determined to be either present or absent. The 79 publications each had their own unique profile. The median number of elements mentioned was 12, although this figure varied widely across the 79 publications. At the low end was a publication that referenced only two elements. At the high end was a publication that referenced 38 elements (22 primary and 16 secondary).

### 3.3 Prevalence of each element

Tables 1 and 2 show the frequency with which each element in the taxonomy was mentioned across the 79 publications. Table 1 includes the 59 elements (25 primary and 34 secondary) that correspond to LHS work, while Table 2 includes the 35 elements (13 primary and 22 secondary) that correspond to enabling conditions. “LHS work” involves learning‐oriented activities that lead to improved patient care, including analysis of clinical data, quality improvement processes, research, and the adoption of evidence‐based practices. In contrast, “enabling conditions” make it possible or easier for people within the organization to carry out LHS work. This category includes elements such as the clinical data infrastructure, the competencies of employees, organizational culture, policies, and institutional investments in LHS work.

**TABLE 1:** Frequency of occurrence of “LHS work” elements (n = 79 publications)

Body of work	Primary element	#	Secondary element	#
Organizational learning, innovation, and continuous quality improvement	Quality improvement processes are standard practice	46	Continuous (or CQ) Improvement processes are used	16
			Rapid (or Rapid‐Cycle) learning processes are used	10
			Plan‐Do‐Study‐Act (PDSA) cycles are used	5
	Learning is done according to particular principles, processes, practices, and/or models	33	Collaborative or team‐based learning	16
			Systems science	8
			The Learning Cycle proposed by Friedman	5
			Collective “sensemaking”	2
			Positive deviance	2
			Triple‐loop learning (“learning how to learn”)	2
			“Emergent learning” or learning in support of “emergent strategy”	0
	Learning is driven/guided by specific goals	13	Equity	6
			Improving the quality of care	4
			Efficiency	3
			Patient safety	2
	Learning takes place throughout the organization	8		
Translating knowledge and evidence into improved practices	Research is translated into practice	26	Research conducted within the organization is translated	7
			Research findings from the literature are translated	2
	The organization adopts or implements evidence‐based treatments	18		
	There is a reciprocal relationship between research and practice	17		
	The organization is systematic in its implementation processes	13	Interventions should be adapted *or* tailored to the specific context	6
			Allows for learning and refinement in implementation	5
			Systematically de‐implements practices that no longer serve the organization	3
			Follows the principles of implementation science	3
			Implements with fidelity	0
Building new knowledge and evidence	The organization builds knowledge or evidence	54		
	The organization conducts “research”	28		
	The research conducted by the organization is practical or needs to balance practical with rigorous	26		
	Findings from the research are shared or disseminated	14	Internally	7
			Externally	6
	Research conducted by the organization answers questions that are directly relevant to the organization	11	Answers questions posed by clinicians (relevant to clinical practice)	5
			Answers questions by organizational leaders (relevant to larger organizational goals)	1
	Data are translated into information	6		
	Internal knowledge and external knowledge are integrated	5		
	The research conducted by the organization is rigorous	4		
Analyzing clinical data	Patient data are captured and organized into a system, which is then used for analysis (research, QI, or other forms of learning)	37		
	Clinical and/or informatics data are used in diagnosing and treating individual patients	34	Clinical decision support systems are in place	22
			Personalized treatment (eg, using genomics data)	12
			Precision medicine	7
	Aggregated clinical data is shared between institutions	14	The clinical data systems of different institutions are networked	3
	Clinical data are analyzed to develop research questions and design studies	3		
Engaging clinicians, patients, and other stakeholders	Patients and family members are actively engaged	40	… engaged in the learning process	18
			… engaged in clinical decision making	13
	Stakeholders (beyond researchers) are engaged in the learning process	37	Stakeholders from within the organization (beyond researchers) are engaged in the learning process	20
	Community members or community‐based organizations are engaged	17	… engaged in the learning process	4
			… engaged in improving the organization	0
	Clinicians are actively engaged in research	7		
	Payors are engaged in the learning process	1		

*Note*: Elements mentioned in at least 20 publications are highlighted in dark yellow. Elements mentioned in 10‐19 publications are highlighted in light yellow.

**TABLE 2:** Frequency of occurrence of “Enabling Condition” elements (n = 79 publications)

Enabling condition	Primary element	#	Secondary element	#
Workforce skilled for LHS work	Employees have the skills and knowledge necessary for LHS work	23	Able to access and analyze clinical data	4
	Organization provides training to employees on LHS competencies	14	Training on quality improvement	7
			Training on research methods	5
			Training on informatics	2
Data systems, informatics technology, and resources are in place	Appropriate informatics technology and resources are in place within the organization	31		
	Clinical data systems and repositories meet rigorous standards	30	Privacy	12
			Quality	10
			Reliability	6
			Validity	3
			Completeness	1
	Data systems are designed strategically anticipating the kinds of research that will be conducted	12		
	Specific fields are included in the EMR to allow for LHS research	12	Patient‐centered outcomes	8
			How care was delivered to each patient (beyond ICD‐10 treatment codes)	0
			Patient feedback (eg, satisfaction ratings)	0
	Aggregated clinical data are made available so that a wide range of learners within the organization can use it for analysis	8		
The organization invests resources in LHS work	Organizational policies incentivize LHS activities	19	Policies incentivize learning	6
			Policies incentivize research	4
			Policies incentivize translation	2
			Policies incentivize patient engagement	0
	The organization invests its own funds to conduct research	9		
	The organization employs embedded researchers (researchers with LHS competencies who carry out studies that address the interests of clinicians and administrators)	6		
	Organization has a dedicated center or institute that provides focus and leadership for LHS work	5		
Supportive organizational culture	Organizational culture emphasizes and supports learning	46	Transparency is valued by the organization	24
			Collaboration (or team‐based learning) is valued	19
			Culture facilitates trust‐building	17
			Integrity is valued by the organization	8
	Learning is championed by organizational leaders	24	Clinicians are encouraged to conduct research	3
			All employees are expected to be active learners	2

*Note*: Elements mentioned in at least 20 publications are highlighted in dark yellow. Elements mentioned in 10–19 publications are highlighted in light yellow.

Within Tables 1 and 2, the elements are further divided into five “bodies of work” and four categories of “enabling condition.” The five bodies of work are:

1. Organizational learning, innovation, and continuous quality improvement that leads to improved patient care;
2. Identifying, critically assessing, and translating knowledge and evidence into improved practices;
3. Building new knowledge and evidence around how to improve healthcare and health outcomes;
4. Analyzing clinical data to support learning, knowledge generation, and improved patient care; and
5. Engagement of clinicians, patients, and other stakeholders in processes of learning, knowledge generation, and translation.

The four enabling conditions are:

1. The organization has a critical mass of employees with the skills and knowledge necessary for LHS work;
2. Data systems, informatics technology, and resources are in place within the organization to support analyses of clinical data that address the organization's learning questions;
3. The organization invests resources that are sufficient to carry out the different bodies of LHS work; and
4. There is a supportive organizational culture with norms, policies, and visible leadership that support LHS work.

Table 1 shows that 12 of the “LHS work” elements (10 primary and 2 secondary) were mentioned in at least 20 publications (one‐fourth of the sample). Three of these were mentioned in at least 40 publications (half the sample): “Organization builds knowledge or evidence,” “Quality improvement processes are standard practice,” and “Patients and family members are actively engaged.” Four others were mentioned by at least 30 publications: “Stakeholders (beyond researchers) are engaged in the learning process,” “Patient data are captured and organized into a system, which is then used for learning,” “Clinical and/or informatics data are used in diagnosing and treating individual patients,” and “Learning is done according to particular principles, processes, practices and/or models.”

Table 2 shows that the most frequently mentioned “enabling condition” elements were: “Organizational culture emphasizes and supports learning,” “Appropriate informatics technology and resources are in place within the organization,” and “Clinical data systems and repositories meet rigorous standards.” Each of these was mentioned in at least 30 publications.

### 3.4 Domain‐level frequencies

**FIGURE 3:**
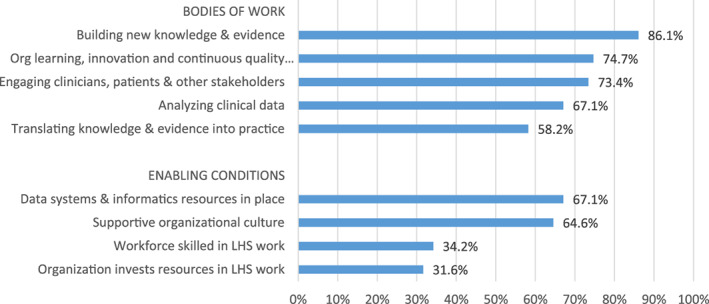
Percentage of publications that address each domain

Figure 3 presents a higher‐order view of which aspects of LHS were emphasized in the sample of 79 publications. This figure reports the percentage of publications that mentioned at least one element within each of the five bodies of work and four enabling conditions. Four of the five bodies of work were included in at least two‐thirds of the publications. The most widely referenced body of work was “building new knowledge and evidence” (86.1%), while the least referenced was “translating knowledge into practice” (58.2%).

Among the four enabling conditions, two were referenced by approximately two‐thirds of the publications: “data systems and informatics resources in place” and “supportive organizational culture.” The remaining two enabling conditions (“workforce skilled in LHS work” and “organization invests resources in LHS work”) were each referenced in only about one‐third of the publications.

This domain‐level grouping of elements allowed us to also assess the degree to which the publications were comprehensive in covering the five bodies of work and four enabling conditions in their descriptions of LHS. The majority of publications referenced either all five bodies of work (23 publications or 29%) or four of them (21, or 27%). In contrast, 17 publications (22%) focused on only 1 or 2 bodies of work. The publications were less comprehensive in referencing enabling conditions. Only 11 (14%) referenced all 4 enabling conditions and another 10 (13%) referenced 3 of them. The majority (52 or 66%) referenced only 1 or 2 enabling conditions. Six publications did not mention any enabling conditions.

## 4 DISCUSSION

### 4.1 Unpacking the LHS construct

This study used a targeted review and analysis of the LHS literature to identify the work that healthcare organizations are doing or should be doing on the way toward becoming an LHS. Our first finding involved the size and diversity of principles, practices, and conditions that researchers point to when describing LHS. In order to capture the full range of LHS‐related issues raised in this sample of publications, we needed to create an extensive taxonomy with 38 “primary” elements, 16 of which are further specified with “secondary” elements. Tables 1 and 2 show that 6 of the 59 “secondary” elements in the taxonomy were anticipated to be relevant, but were not actually mentioned in the sample of publications. We have elected to retain these six “null” elements in the taxonomy because we believe they may be applicable in future LHS publications.

A second overarching result was that the elements associated with LHS fall into two distinct categories: LHS work (ie, specific learning‐oriented activities carried out with specific intents and according to specific principles) and enabling conditions that need to be present within a healthcare organization in order for LHS work to be carried out. We further categorized the elements into five “bodies of work” (continuous learning and quality improvement, building knowledge and evidence, translating knowledge into practice, analyzing clinical data, and engaging stakeholders) and four “enabling conditions” (workforce skilled for LHS work, relevant data systems and informatics resources in place, dedicated investments in LHS work, and a supportive organizational culture).

### 4.2 A framework for understanding and implementing LHS

**FIGURE 4:**
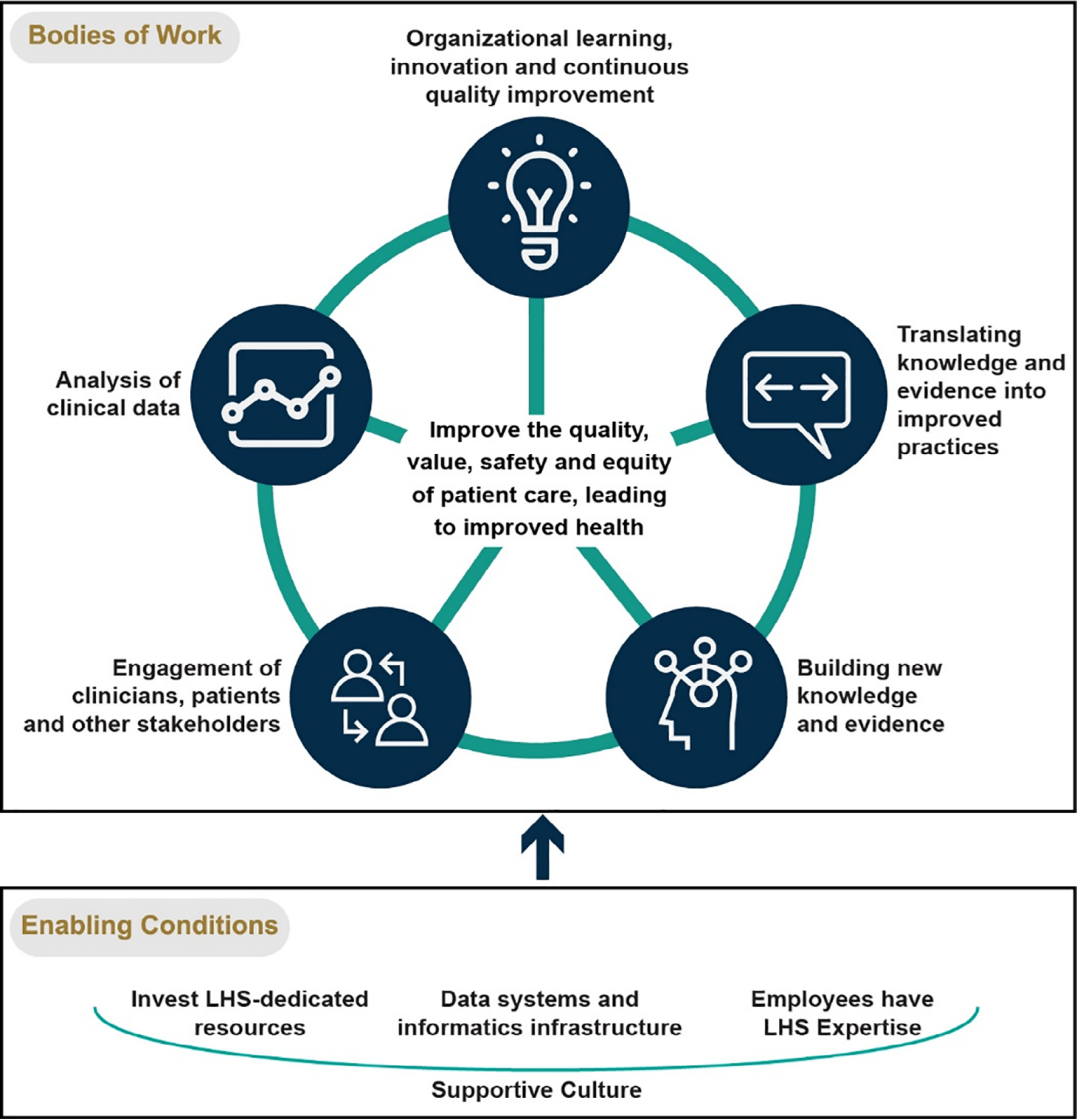
Learning Health Systems Consolidated Framework (LHS‐CF)

We believe that these five bodies of work and four enabling conditions serve as a useful framework for organizing LHS research, as well as the planning, budgeting, management, leadership, and evaluation that is required to transform a healthcare organization into an LHS. Figure 4 shows the conceptual linkages among and between the five bodies of work and four enabling conditions. We refer to this conceptual map as the “Learning Health System Consolidated Framework” (LHS‐CF) because it is a consolidation of the different ways that LHS has been described, studied, and promoted in the scientific literature.

The top portion of the LHS‐CF figure shows that the five bodies of work are interconnected and mutually reinforcing. The bottom portion shows that LHS work is facilitated by (and depends upon) the four enabling conditions.

It is important to point out that LHS‐CF is consistent with the current NAM definition of LHS, while also adding a conceptual mapping of the different areas of work that are covered within the definition. Perhaps most importantly, LHS‐CF makes a clear distinction between the work that occurs within an LHS and the conditions that need to be in place to support that work. Moreover, LHS‐CF emphasizes the fundamental importance of a supportive organizational culture by making it the foundation of the figure. This was the most widely referenced enabling condition within our sample of publications, which is consistent with the prominence of organizational culture within theories and frameworks for implementing complex interventions.^92^

At the core of LHS‐CF is the fundamental purpose of LHS work: “to improve the quality, value, safety and equity of patient care, leading to improved health.” This intent is drawn directly from the characterizations of LHS that have been championed by NAM and AHRQ over the past 13 years. The publications reviewed in our study affirmed that the LHS concept is concerned primarily with improving the care delivered by health systems to their own patients, with the collateral benefit of contributing new knowledge and innovation that helps other organizations also improve their care. We have added an emphasis on equity because this is increasingly being recognized as a critical goal for health systems with regard to patient care and population health. Six publications in our sample explicitly mentioned equity as a goal that should guide the learning that occurs within an LHS. Equity has become even more prominent among LHS articles published since January 2020. For example, Allen et al.^93^ included equity as an outcome in their LHS logic model in a manuscript published in 2021. They define equity as “fairness in processes, outcomes and relative costs.” (7)

### 4.3 Which LHS elements are crucial?

The second aim of the study was to determine, which forms of LHS work and enabling conditions have been emphasized in the literature, with the expectation that these findings can provide guidance to health system leaders in determining what needs to be built or enhanced within their organizations in order to qualify as an LHS. Toward that end, Tables 3 and 4 highlight the LHS work elements and enabling conditions in the taxonomy that were referenced by at least 10 of the 79 publications.

**TABLE 3:** LHS Work Elements referenced in at least 10 publications (with some synthesis)

Bodies of LHS work	Specific forms of work frequently mentioned in the literature
Organizational learning, innovation, and continuous quality improvement which leads to improved patient care	Structured, goal‐oriented learning is integrated into operations. Learning processes are explicitly designed to improve quality, safety and value of care, and to enhance efficiency of operations. Quality improvement processes, including continuous quality improvement and rapid‐cycle learning, are routinely employed throughout the organization. Collaborative or team‐based learning is practiced.
Identifying, critically assessing, and translating knowledge and evidence for improved patient care	Relevant internal and external research findings are identified and translated into treatments and practices that improve patient outcomes and organizational performance. The organization systematically adopts and implements evidence‐based treatments.
Building new knowledge and evidence to improved patient care and health outcomes	The organization conducts research (beyond quality improvement) to answer questions that relate directly to the organization's goals and issues regarding patient care. Research conducted by the organization balances rigor with practicality and cost‐effectiveness. Findings from research are shared/disseminated
Analysis of clinical data to support learning, knowledge generation, and improved patient care	Patient data are captured and organized into a system so that it can be analyzed for research, quality improvement, and other forms of learning. Employees throughout the organization routinely access and analyze clinical data for research and learning. Clinical and/or informatics data are used in diagnosing and treating individual patients. Clinical decision support systems are in place and routinely used. Clinical data are analyzed to support personalized treatment and/or precision medicine.
Engagement of clinicians, patients, and other stakeholders	Stakeholders from throughout the organization (including practicing clinicians) are directly engaged in the learning process. Patients and family members are actively engaged in learning and/or clinical decision‐making. The organization reaches out to external partners with a stake in improving patient care and provides meaningful forums for influencing the learning agenda.

**TABLE 4:** Enabling conditions referenced by 10 or more publications

Enabling conditions of an LHS	Specific enabling factors frequently mentioned in the literature
Expertise	Employees throughout the organization have the skills and knowledge to engage in structured learning, quality improvement, data analysis, etc. Organization provides training to employees on LHS competencies (eg, quality improvement, research methods, and analysis of clinical data).
Data systems and informatics infrastructure	Appropriate informatics technology and resources are in place within the organization. Clinical data systems are designed strategically (anticipating the questions that investigators will bring and the analyses to be conducted). Clinical data systems and repositories meet rigorous standards, especially privacy, quality, and reliability.
Investment of LHS‐dedicated resources	Organizational policies incentivize LHS activities
Supportive culture	Organizational leaders are active, visible champions of LHS principles and practices. The organization has norms that encourage and support learning, translation of evidence into practice, building knowledge, and patient engagement. Organizational policies and culture promote transparency, integrity, and trust‐building. Collaboration and/or team‐based learning are valued.

Tables 3 and 4 provide a top‐level view of what aspects of LHS have been emphasized in the literature, but we do not want to imply that these are lists of “essential” forms of LHS work and enabling conditions. For any given health system, some elements will be more relevant than others.

In addition, we believe that some of the less frequently mentioned elements in the framework (not included in Tables 3 and 4) are quite important for health system leaders to incorporate into their planning and decision making. The most important of these in our view is “the organization invests resources in research that addresses its priority questions” (referenced in nine publications). Along similar lines, we would also highlight “The organization employs embedded researchers (researchers with LHS competencies who carry out studies that address the interests of clinicians and administrators),” which was also referenced in nine publications. We believe that any health system leader considering the idea of transforming their organization toward an LHS needs to be fully cognizant that this involves extensive and sophisticated new work requiring significant monetary investments, hiring of new staff with specialized skills, and re‐allocation of effort toward continuous learning and knowledge generation.

One other important qualification in using Tables 3 and 4 as guides for implementing LHS has to do with vagueness of some of the elements. While some of the LHS work elements and enabling conditions are specific in nature (eg, organization provides training), others reflect general principles (eg, research is translated into practice). Thus, these lists provide only global guidance to health system leaders. Further planning is required to operationalize these elements and to prioritize, which are most critical to the organization's mission and vision.

### 4.4 Limitations of the study

The LHS‐CF model was derived from a comprehensive review and systematic analysis of literature that describes the work carried out by healthcare organizations that are operating within the LHS paradigm. The sample included peer‐reviewed journal articles, workshop proceedings, and book‐length monographs. However, the search was limited to publications included in the PubMed database. Other documents describing the LHS concept certainly exist. It is possible that they point to features of the LHS concept not included in our taxonomy. Similarly, the larger literature base might have a different frequency distribution of LHS elements than was found in the 79 publications reviewed in this study. Our hypothesis, which warrants testing, is that the elements found to be most frequent in our sample will also be most prominent within the broader literature.

A second limitation to our approach is that we focused on the proportion of publications mentioning an element as the basis for assessing the centrality of the element to the LHS construct. Some elements (especially involving clinical data systems) might be of intense interest to a relatively large number of academics who publish LHS‐related articles, but may not be the most essential elements from the standpoint of those who have the greatest expertise or authority with regard to defining and implementing the LHS concept. In addition, it may be that the scientific literature on LHS under‐recognizes and/or under‐emphasizes some of the elements that are most essential to creating and sustaining a high‐functioning, high‐payoff LHS. One example from our standpoint is “the organization invests its own funds to conduct LHS research.”

A third limitation is the evolution of the LHS concept over time. This study was based on articles listed in PubMed as of January 2020. The literature on LHS is expanding rapidly. As it does, there may be significant shifts in the relative frequency of elements within the taxonomy.

## 5 CONCLUSION

Our intent with this study was to provide the leaders of health systems with a clearer view of the specific types of work that need to be launched and supported in order to operate according to the principles of an LHS. With this clarity comes a greater appreciation of how the organization needs to evolve and what sorts of investments will be required.

We believe that LHS‐CF can provide useful guidance for the implementation of LHS within health systems ‐ by pointing to the range of activities, approaches, tools, conditions, and principles that are associated with the concept. On the other hand, becoming an LHS involves specific decisions about concrete work. Each health system needs to operationalize LHS in ways that fit with its own mission, values, and needs.^93^, ^94^ More specific research, especially case‐study research, is needed to inform the operationalization of LHS.

We expect that the LHS‐CF model will cause some health system leaders to regard the LHS concept as a more ambitious endeavor than was implied by the shorter definitions of LHS proffered by NAM and AHRQ. Many healthcare organizations will need to make major enhancements to their clinical data system in order for employees to be able to readily access the data and conduct analyses that answer their learning questions (as opposed to simply managing individual patients). The LHS‐CF model also makes it clear that analyzing clinical data and building knowledge requires professional staff with specialized skills. Hiring these individuals will require significant financial investments and may divert resources away from clinical operations. In theory, building out the capacity to conduct LHS work will return dividends with regard to more efficient care delivery, but this calculus presumes a long time horizon for investment decisions.

The situation is different for academic health systems. They begin the LHS journey with considerable capacity in research and informatics, and typically have clinical data systems that are well suited to learning‐oriented analyses. To the extent that clinicians in the health system are actively engaged in the conduct of research, there might be direct routes for translating knowledge into practice, and more particularly for implementing evidence‐based interventions studied within the institution. On the other hand, many academic health systems have chasms between the research and clinical enterprises, which limits the amount of translation that occurs.^40^

Regardless of what type of healthcare organization, becoming an LHS will be a challenging journey. All healthcare organizations will need to develop new competencies, invest in new infrastructure, and engage in new practices in order to meet the expectations associated with the LHS concept.

## CONFLICT OF INTEREST

The authors report no conflicts of interest.
